# Descriptive Study of Gender Dysphoria and Sexual Behavior in a Disorder of Sex Development Group

**DOI:** 10.3389/fpsyg.2022.652030

**Published:** 2022-10-07

**Authors:** Cesar Augusto Bridi Filho, Saulo Batinga Cardoso, Bianca Machado Borba Soll, Marcelo Fröelich Noal, Karine Schwarz, Tiago Elias Rosito, Nicolino Cesar Rosito, Maria Inês Rodrigues Lobato

**Affiliations:** ^1^Department of Psychiatry and Legal Medicine, Hospital de Clínicas de Porto Alegre (HCPA), Federal University of Rio Grande do Sul (UFRGS), Porto Alegre, Brazil; ^2^Department of Pediatric Surgery and Department of Urology, Hospital de Clínicas de Porto Alegre (HCPA), Federal University of Rio Grande do Sul (UFRGS), Porto Alegre, Brazil

**Keywords:** gender dysphoria, disorder of sex development, DSD, sexual behavior, sexuality

## Abstract

Disorder of Sex Development (DSD) refers to a heterogeneous group of congenital conditions in which chromosomal, gonadal, and anatomical sex are atypical. Typically, the diagnosis is made at birth or infancy and interventional actions are necessary in many cases. The repercussions in adult life, more specifically in the field of sexuality, have not been not widely studied yet. This study shows research data that seek to identify in a group of individuals with DSD (XX DSD, XY DSD, Chromosomal DSD), who are being monitored in the departments of pediatric surgery and urology of a hospital in the period from 2000 to 2019, and to verify the consequences on sex life after puberty. The sample has 16 participants (7 XY DSD, 4 XX DSD, and 5 Chromosomal DSD), aged between 16 and 50 years, single, with high school education, residents of the state capital and countryside of the state. The results depict the presence of a case of Gender Dysphoria; postponement of sexual debut for almost 3 years compared to the national average; a single sexual relationship for those who have already had a sexual experience with penetration; penis size below the general population average; presence of masturbatory activity in most participants. The presence of sexual attraction and masturbation indicate sexual desire. The group has a late onset of sexual life (almost 3 years after the national average). A recurrent feature in this group is that, even having already performed a penetrative sexual act, there is no continuation in their sexual life. The main noticeable aspect about Gender Dysphoria is the presence of only one case of incongruence in the Chromosomal DSD group. The limited sample prevents us from sufficient statements for generalization.

## Introduction

Disorder of Sex Development (DSD) corresponds to the heterogeneous group of congenital conditions in which chromosomal, gonadal, and anatomic sex development are atypical (Scolfaro et al., 2005; Lee et al., 2006; Hughes, 2015). DSDs were categorized into three main subgroups, according to karyotype: XX, XY, and Sex Chromosome for mosaic karyotypes (Romao et al., 2012). With varied incidence in different populations (Scolfaro et al., 2005; Lee et al., 2006; Romao et al., 2012; Hughes, 2015, 500–5,5,000), it is considered a rare phenomenon (Lee et al., 2016). In general, the diagnosis is made at birth or infancy and interventional actions are necessary in many cases, despite the considerable existing controversy, especially nowadays. Some types of atypical genitalia at birth may be considered DSD, together with all chromosomal, gonadal, and genital sex discrepancies.

Non-interventional or clinically based studies present the long-term outcomes of hormone therapy, surgical intervention, social and psychological support to patients with DSD conditions from adolescence onwards (Röhle et al., 2017; Baudewijntje et al., 2018; Rapp et al., 2018). For people with DSD, quality of life, sexual aspects, physical and psychological wellbeing, as well as satisfaction with the treatment (hormonal, surgical, psychological) are predictors of good global development (Ernst et al., 2019).

Sexuality is one of the elements that constitute human development and, in people with DSD, it is one of the elements that is directly affected. It involves elements, such as social image, body image, degree of identification with the assigned gender, fertility, and sexual behavior (McCabe et al., 2010; Monteiro Pascoal et al., 2015; Schweizer et al., 2017).

According to the DSM-5 (American Psychiatric Association, 2013), Gender Dysphoria (GD) as a general descriptive term, refers to the affective/cognitive incongruence of an individual with the sex designated, although it is more specifically defined when used as a diagnostic category. Individuals with Gender Dysphoria present marked inconsistencies between their assigned gender (the birth gender) and the experienced/expressed gender. The diagnosis comes from the existence of distress between the expressed and the designated gender, and not just the differentiation between them. The DSD is one of the GD diagnosis specifiers, however, it does not describe the specific characteristics for this group, taking into account the multiple variables throughout development (Kraus, 2015).

The sexual response (including physiological responses to desire, arousal, and orgasm) expressed through sexual behavior is associated with positive or negative affection and general wellbeing about oneself and the body (Peterson and Janssen, 2007; Poeppl et al., 2016). Many people with DSD conditions are less sexually active than the general population, they do not seem satisfied with their sex lives, and experience a wide range of sexual problems (Kreukels et al., 2019). Men with DSD show dissatisfaction with the penis length, ejaculation, satisfaction with the external genitalia, and frequency of sexual activity, with different levels of satisfaction between subgroups (Van Der Zwan et al., 2013). Women with DSD may experience difficulties with the body and sexual activity, as well as the size and width of the vagina, the size of the clitoris, or fears for intercourse, the percentage varying by subgroup (Kleinemeier et al., 2010; Kreukels et al., 2019).

The aim of this study is to investigate aspects related to sexual behavior—gender dysphoria and aspects related to sexual activity—in a group of 16 adolescents and adults in a systematic follow-up at the HCPA pediatric surgery and urology clinic (Table 1). These people underwent some surgery and/or received systematic follow-up within this specific unit of the Hospital between 2000 and 2019. DSD core diagnoses may coexist with other associated diagnoses.

**Table 1:** Division by groups.

Division by groups					
		Frequency	Percentage	Valid percentage	Cumulative percentage
Valid	Chromos DSD	5	17.2	31.3	31.3
	XX DSD	4	13.8	25.0	56.3
	XY DSD	7	24.1	43.8	100.0
	Total	16	55.1	100.0	

## Procedures

All patients had records and clinical follow-up at the HCPA Pediatric Surgery and Urology Outpatient Clinic, with a confirmed diagnosis of DSD. The interviews were carried out in the hospital premises, after the annual/biannual clinical care provided to the patients. All protocols were applied during a single session, preceded by the reading of the informed consent, presentation and description of the procedures to be performed, with the purpose of evaluating the general conditions and aspects of the participants’ body image and sexuality. The group consisting of DSD/Chromosomal people underwent the same protocol, but online, *via* forms, due to the restrictive conditions of the SARS-COVID-19 pandemic during the collection period.

The results described are part of a wide research which involves ten protocols for the global assessment of patients. The protocol includes the sociodemographic assessment of the Brazilian Institute of Geography and Statistics (IBGE); Quality of Life (WHOQOL-BRIEF); Anxiety and depression (DASS22); Rosenberg Self-Esteem Scale (EAR); Body Image (Body Image Scale—BIS); Body Satisfaction (Utrecht Scale); Male/Female Sexual satisfaction (MSQ and FSQ); ICD-11 inventory of the study in the Brazilian field (Soll et al., 2018; Lobato et al., 2019); Kinsey Scale of Self-Denomination of Sexual Orientation; Descriptive questionnaire about the participant’s body and sex life. In this study, data referring to the presence or not of Gender Dysphoria, MSQ, and FSQ Scale were selected, in addition to aspects reported by patients during the questionnaire about their sexual life.

## Results

### Sociodemographic Profile

In the sociodemographic characterization of patients (Table 2), age ranged from 18 to 50 years, with an estimated mean of 22.4 (*sd* = 8.7) years. When this piece of information was compared between groups, the XY DSD cases (18.4 ± 2.7) concentrated significantly younger ages (*p* = 0.035). Most patients live outside the state capital city 68.8% (*n* = 11) and a similar situation was observed in the three groups (*p* = 0.622). Only 31.3% of the patients live in the same municipality as the Hospital where the research was carried out, a factor that hinders access to or return to appointments.

**Table 2:** Absolute and relative distribution for sociodemographic characterization and measures of central tendency and variability for age, over the total sample and per group.

Variables	Total sample (*n* = 16)^*^		Groups^**^						
			CHROMOS DSD (*n* = 5)		XX DSD (*n* = 4)		XY DSD (*n* = 7)		*p*
	*n*	%	*n*	%	*n*	%	*n*	%	
**Age (years)**									0.035^B^
Mean ± standard deviation (amplitude)	22.4 ± 8.7 (16–50)		25.0 ± 6.1 (18–32)		26.0 ± 16.0 (17–50)		18.4 ± 2.7 (16–24)		
Median (1st – 3rd quartile)	19 (18–25)		25 (19–31)		19 (17–42)		18 (16–19)		
**Education**									0.731^A^
Elementary school	3	18.8			1	25.0	2	28.6	
High school	10	62.5	3	60.0	3	75.0	4	57.1	
Tertiary education	3	18.8	2	40.0			1	14.3	
**Place of residence**									
State capital city	5	31.3	2	40.0	1	25.0	2	28.6	0.598^A^
Region	11	68.8	3	60.0	3	75.0	5	71.4	
**Religion**									0.096^A^
Catholic	4	25.0			3	75.0	1	16,7	
Evangelic	7	43.8	2	40.0	1	25.0	4	66.7	
Afro-Brazilian	1	6.3					1	16.7	
NR	4	25.0							
**Importance of religion** ^A^									0.202^A^
Very important	6	37.5	2	40.0	2	50.0	2	28.6	
Not very important	7	43.8	2	40.0	1	25.0	4	57.1	
Not important	3	18.8	1	20.0	1	25.0	1	14.3	

* Percentages obtained based on the total cases with valid answers in the sample.

** Percentages obtained based on the total number of valid responses in each group.

A Fisher’s Exact Test.

B Kruskal–Wallis Test—Post-Hoc Dunn.

Respondents’ education level is high school (HS), complete or nearing completion, reaching 62.5% (*n* = 10). This same characteristic was maintained in groups XX DSD, 75% (*n* = 3) and XY DSD, 57.1% (*n* = 4), and Chromosomal DSD, 62.5% (*n* = 10; *p* = 0.731).

Evangelical religion had the highest frequency in the sample, 43.8% (*n* = 7); as well as, in the XY DSD group, 66.7% (*n* = 4). However, among the XX DSD patients, the Catholic religion predominated, 75% (*n* = 3; *p* = 0.096). When asked about the importance of religion, 47.4% (*n* = 9) mentioned “it is not very important.” This result was repeated in the XY DSD group, 57.1% (*n* = 4), but not in the XX DSD group, where 50% (*n* = 2) declared religion as “very important.”

### Sexuality and Identity

In the information regarding surgical procedures (Table 3), most patients/medical records 75% (*n* = 12) confirmed some surgical procedure related to the genitals. This result was repeated in the XX DSD groups, 100% (*n* = 4), and in the XY DSD, 100% (*n* = 7). Regarding the patients in the Chromosomal DSD group, 80% (*n* = 3) did not undergo a surgical procedure. The following urogenital interventions were performed as recorded in the medical record: 46XY DSD Group—Hypospadias Correction (Lee et al., 2016); Vaginoplasty (Hughes, 2015); Cliteroplasty (Hughes, 2015); Hydrocelectomy (Hughes, 2015); Orchidopexy (Scolfaro et al., 2005); Urethral fistula correction (Lee et al., 2006); Meatotomy (Hughes, 2015) and Oophorectomy (Hughes, 2015). In the 46XX DSD Group: Urethroplasty (Hughes, 2015); Panhysterectomy (Hughes, 2015); Hypospadias correction (Hughes, 2015); Correction of penile curvature (Hughes, 2015); Vaginoplasty (Scolfaro et al., 2005); Cliteroplasty (Scolfaro et al., 2005); Urethroplasty (Hughes, 2015); Chromosomal Group: Hypospadias Correction (Lee et al., 2006); Urethroplasty (Lee et al., 2006); Orchiopexy (Lee et al., 2006); Scrotoplasty (Hughes, 2015); Testicular Prosthesis (Hughes, 2015); Meatotomy and Dilation of the Urethra (Hughes, 2015).

**Table 3:** Data referring to diagnosis, age, assigned gender, expressed gender, surgical interventions, penis size, and sexual intercourse (divided in groups).

Partic.	Group	Diagnosis	Age	Assigned gender.	Expressed gender	Num. cirurg	Cirurg	Penis size (cm)	Sexual interc.	First. interc
1	DDS46XY	Def. 5 alpha-reductase	18	M	M	1	1	7	1	16y.o.
2	DDS46XY	PAIS	19	M	M	3	1,6,7	11	1	18y.o.
3	DDS46XY	Def. 5 alpha-reductase	17	M	M	1	1	7		
4	DDS46XY	PAIS	16	M	M	2	1,5	17		
5	DDS46XY	PAIS	19	M	M	3	1,5,6	11	1	19 y.o
6	DDS46XY	Primary hypogonadism—Gene Variant DHX37	16	M	M	2	4,5	8		
7	DDS46XY	PAIS	24	W	W	3	2,3,1			
8	DDS46XX	CAH (21-hydroxylase deficiency)	50	M	M	4	9,10,1,11	NR		
9	DDS46XX	CAH (21-hydroxylase deficiency)	17	W	W	2	2,3			
10	DDS46XX	Turner Syndrome—Hypothyroid with elevated TSH	19	W	W	2	2,3		1	18 y.o
11	DDS46XX	CAH (21-hydroxylase deficiency)	18	W	W	4	2,3,9,12			
12	DDSCrom	Turner Syndrome	32	W	W	0	/			
13	DDSCrom	Turner Syndrome	30	W	W	0	/			
14	DDSCrom	Turner Syndrome	25	W	Mt^*^	0	/			
15	DDSCrom	Mixed Gonadal Dysgenesis	20	M	M	4	1,7,9,16	8	1	18 y.o
16	DDSCrom	Mixed Gonadal Dysgenesis	18	M	M	5	1,5,9,14,14	NR		

Surgical interventions: 1—Correction of Hypospadias; 2—Vaginoplasty; 3—Cliteroplasty; 4—Hydrocelectomy; 5—Orchidopexy; 6—Urethral fistula correction; 7—Meatotomy; 8—Oophorectomy; 9—Urethroplasty; 10—Panhysterectomy; 11—Correction of penile curvature; 12—Correction of hypertrophy of the labia minora; 13—Scrotoplasty; 14—Testicular Prosthesis; 16—Dilation of the Urethra.

* Expressed gender: “Trans Man.” NA: “Did not answer.”

The mean age for the first surgery was 10.6 years (sd = 7.1) years, with the minimum age being 2 months (0.17 years) and the maximum being 10 years and 3 months (10, 3 years). When this piece of information was compared between groups, there is evidence that the mean in the XY DSD group (13.5 ± 6.7) was significantly later (*p* = 0.011) than the estimate in the XX DSD group (5.3 ± 4.5). Considering the number of procedures, out of the total sample, the median was two procedures, with a minimum of one and a maximum of four surgeries. When this piece of information was evaluated between groups, the median of two procedures was observed in groups XX DSD and XY DSD. In the Chromosomal DSD group, out of the four patients, only one answered this question, informing three procedures.

When comparing the sex designated at birth, men comprise a group of nine (56.3%) participants and women are seven participants (43.8%) in the sample. However, when compared to the identified gender, all nine men (100%) identify themselves as men, and among women, six of them identify themselves with the female gender (87.71%). One of the participants who was assigned female at birth (DSD Chromosomal group) identifies as a “trans male.” Nevertheless, in this case, the participant does not present criteria for the diagnosis of “Gender dysphoria.”

One of the participants (number 7) was assigned as a “woman” at birth, but at puberty, she presented a penile growth. However, after surgical interventions, she maintained her identity in congruence with the sex assigned at birth. One participant (number 8) was designated as a “male” at birth and developed his identity as a male, also showing no incongruity throughout his development. In both cases, they do not report sexual life with penetration or masturbation.

All participants, by self-declaration through the Kinsey Scale (Heterosexuality/Homosexuality/Bisexuality), expressed their sexual orientation. Among the 12 heterosexuals, four are from the DSD Chromosomal group, two from the XX DSD, and six from the XY DSD group. Homosexuality is present in one man from the Chromosomal group and Bisexuality was declared by three women from the XX DSD group.

As for the type of relationship (single/dating/married/other), 75% (*n* = 12) of the cases reported being single, and this result was maintained in relation to the groups (*p* > 0.999). Masturbation as a sexual practice was confirmed by 62.5% (*n* = 11) of the patients, a result that also stood out in the XX DSD, 75% (*n* = 3) and XY DSD, 71.4% (*n* = 5) groups.

Intercourse with penetration was confirmed by 33.2% (*n* = 5). When this information was compared between the groups, it was found that, in the DSD Chromosomal and XX DSD groups, the cases that confirmed intercourse with penetration, with proportions of 20% (*n* = 1) and 25% (*n* = 1), respectively. Among the XY DSD cases, this proportion that confirmed a sexual life with penetration was 42.8% (*n* = 3).

In a stratification by diagnosis, there is no significant representation for “penetrative sex life” and diagnosis. Participants 2 and 5 in the DSD46XY group who reported having had penetrative sexual intercourse have a diagnosis of PAIS and participant 1, in the same group, has a diagnosis of 5α-reductase deficiency. None of the people with CAH in the DSD46XX group or with Turner Syndrome in the DSDCROM group reported penetrative sex. Masturbation presents itself in a similar way in terms of diagnosis. In the DSD46XY group, both people with PAIS and 5α-reductase deficiency report the presence of masturbation, as did 3 people with CAH in the DSD46XX group. In the DSD CROM group, only participants with Mixed Gonadal Dysgenesis report masturbatory practice, correlating with body image (penis/clitoris), what is possible to see is a means of 3 points (satisfactory) for the body, and only 4 participants (25%) “very satisfied” with their genitals, and the same proportion (25%) were “very dissatisfied” with their genital appearance. Body image may be an inhibiting factor of sexual practice in this group.

Among the 5 patients who confirmed having intercourse with penetration, for the total sample, the mean age at first intercourse was 17.7 (*sd* = 1.1) years, and in the XX DSD group, the only case reported an age of 18 years, while in XY DSD the mean was 17.6 (*sd* = 1.2) years. Regarding the number of intercourses with penetration, the number of only one intercourse (100%—*n* = 5) stood out for the five cases. When asked if they recognized any sexual dysfunction, out of the five patients who confirmed intercourse with penetration, 100% (*n* = 5) reported that they did not recognize it, and this result was found to be present in all groups.

### Sexual Act

The result of responses to the five domains (erectile function, orgasm, sexual desire, sexual satisfaction, and general satisfaction) evaluated in the Male Sexual Quotient (MSQ), were answered by eight participants from the Chromosomal DSD and XY DSD groups. One of the participants declined to respond. Among the reasons for not having intercourse with penetration, “did not find the ideal partner (Romao et al., 2012),” feelings of fear (Lee et al., 2006), anxiety (Scolfaro et al., 2005), and shame (Romao et al., 2012) were described.

In this assessment of the stages that involve the sexual act from desire (beginning) to its resolution (orgasm), only the five participants who had a sexual life with penetration scored beyond the domain “desire (sexual interest).”

Regarding the data referring to the SQ scale, in the MSQ analyses, in the DSD Chromosomal group, out of the 5 patients in this group, four responded to this scale. The highest mean score occurred in the item “Sexual desire and interest” (2.3 ± 2.6; median = 2.0). As for the lowest means, these occurred in the items “Overall Satisfaction” (1.0 ± 2.0; median = 0.0) and “Self-confidence” (1.0 ± 2.0; median = 0.0). In the MSQ estimates, in the XY DSD group, the highest mean occurred in the question “Sexual desire and interest” (4.2 ± 0.4; median = 4.0), and the lowest mean in the question “Self-confidence” (1.8 ± 1.8; median = 2.0).

In the diagnostic description, there is no difference in the DSD46XY group between PAIS and 5α-reductase deficiency on “sexual desire and interest.” The same assessment is presented by participants with Mixed Gonadal Dysgenesis in the DSD CROM group. People with Turner Syndrome do not score on the MSQ and FSQ scales, except for one participant with a sex life with an increase in stages of sexual intercourse (average of 90% of satisfaction).

Regarding the “self-confidence” aspect, in the DSD46XY group, participants with 5α-reductase deficiency report a total lack of confidence for sexual conquest. When compared, within the group, people with PAIS demonstrate a moderate self-confidence in this item. In the other items, there are no differences between participants with diagnoses in the same DSD46XY group.

When the mean MSQ scores were compared between the Chromosomal DSD and XY DSD groups, statistically significant differences were evidenced, pointing to higher scores in the XY DSD group. Regarding the total MSQ sum, again the mean in the XY DSD group (67.6 ± 33.8; median = 82.0) was higher than the Chromosomal DSD group (49.0 ± 55.1; median = 49.)

In the instrument Female Sexual Quotient (FSQ), the scores referring to the four XX DSD cases, the highest averages were concentrated in the questions “Sexual desire and interest” As for the lowest average scores, these were in charge of the questions “Comfort” (1.9 ± 2.9) and “Orgasm and satisfaction” (1.7 ± 2.9). Considering the estimate of the FSQ sum, the average reached 40.7 (*sd* = 51.4). had intercourse with penetration. Another fact is that satisfaction and orgasm refer to sexual relations with partners and do not consider the pleasure of masturbation.

## Discussion

The construction of sexuality involves many objective and subjective elements. Sexual behavior can be affected by DSD in several ways related to the condition and treatment. Genital ambiguity can cause physical problems, including difficulties with penovaginal intercourse. It can also lead to widespread social stigmatization, undermining self-esteem and leading to avoidance of nudity, sex, and dating (Berenbaum and Meyer-Bahlburg, 2015).

Gender Dysphoria described as low in case reports and follow-up studies reveal that it is most frequently seen among 46, XY non-gonadectomized individuals raised as girls who were exposed to high levels of effective androgens again from puberty (Furtado et al., 2012; Callens et al., 2016). In this group, Gender Dysphoria was non-existent in both XX DSD and XY DSD, regardless of previous treatment. The sample is small and may not be representative of the DSD population. In this case, Gender Dysphoria reaches levels similar to that of the general population, with less than 1% of dysphoric people who present some inconsistency with their gender (Baudewijntje et al., 2018). The only case of incongruity between the sex designated in infancy, and the gender with which this person identifies themselves was in the Chromosomal Group, in which the participant identifies themselves as a “Trans man” and a woman at birth. The other two cases (DSD 46XX and DSD 46XY), even with different designations concerning the genetic aspects, did not present any incongruity or dysphoria in relation to the sex assigned at birth.

In the sample, three XY DSD men, one Chromosomal DSD man, and one XX DSD woman have sex life with penetration. When analyzed in subgroups by diagnosis (PAIS, 5α-reductase deficiency, Mixed gonadal dysgenesis), there is no significant correlation by diagnostic category. The low female sexual frequency, even in a small sample, is in line with the literature, in which female adolescents are less sexually active (Kleinemeier et al., 2010). In the case of XY DSD, the experience of penetrative sex is greater. The average onset of sexual life is at 17 years and 7 months, considered later than the general population, except for one of the participants, who started at the age of 18. In the Brazilian population, about 35% of the adolescents in urban areas have had sexual intercourse before the age of 14 and an average of 15 years in rural areas (Oliveira-Campos et al., 2014; De Sousa et al., 2018). One element that is repeated among the participants is that they all reported having had only one sexual relationship. This may indicate an experimenting relationship just lacking a continuation or interest in sex life for other associated reasons.

In the case of XY DSD men, the average penis size in erection (10.2 cm) is considered below the Brazilian national average (14.5 cm; Gabrich et al., 2007). Male sexual function can be achieved, but there is a possibility of dissatisfaction with the quality of sexual life. Dissatisfaction with penis size can affect self-esteem and quality of life (Kreukels et al., 2019).

The reference to feelings, such as fear, shame, and anxiety, even if not quantified in our population, shows us a constant reality in people with DSD, as reported in previous research: feelings of shame and the experience of stigmatization affect psychological wellbeing. Due to uncertainty about the genital appearance and functional performance, many individuals with DSD fear intimacy and report sexuality-related anxiety and distress, resulting in a tendency to delay or avoid the sexual experience (Cools et al., 2018).

About the sexual act, in particular, even if not all the participants have answered the questionnaires, the 46XY DSD group and the men of the DSD Chromosomal group demonstrated to be interested in a sexual life. However, they do not feel safe about the continuation of the sexual act and have low levels of satisfaction and self-confidence in relation to the sexual act, except for those who have already had sexual experience with penetration. Among the five men who had already had intercourse with penetration, four reported micropenia (by self-reported measurement of the erect penis). None of them have a partner, they are alone/single. Difficulties in ejaculatory control, recognized by the participant, are present in 40% (two participants), however, based on a single sexual relationship, we cannot affirm it is a sexual dysfunction. All of them report good erection ability and maintenance of erection during sexual intercourse. The association of this quotient related to sex life, penis size, and negative feelings (shame, anxiety, and fear) can be factors for late onset and dissatisfaction with sex life. Even among those who have had penetrative sexual activity, all of them report having had only one sexual relationship, without a continuation of the sexual function in their lives. This condition of “experimentation” with a single sexual relationship can interfere with the judgment of their performance and make it difficult to include sexual activity in future relationships.

Among women, only two from the XX DSD group showed sexual interest through the questionnaire. The others did not respond to the in-person or online questionnaire. Among these two, only one woman reported having had a penetrative sex life, but with only one sexual relationship. Among them, the religion factor linked to the Evangelical and Catholic Churches (in general, more restrictive in terms of sexual practice) is very important in their lives and can be an inhibiting factor in sexual behavior and thoughts, even in the field of research.

## Conclusion

Preliminary and initial research data point to a scenario of the construction of sexuality in a specific group of people with DSD who are being monitored at the pediatric surgery and urology clinics of the Hospital de Clínicas de Porto Alegre. It is an exploratory work that depicts the development of the sexual life of people diagnosed with DSD, who have undergone some surgical intervention on their genitals.

This study, aligned with the world literature, emphasized a late onset of sexual life, almost 3 years after the national average. A recurrent feature in the report of this group is that, even having already performed the sexual act with penetration, there is no continuation in their sexual life. This act can be perceived more as an experimentation than an ongoing sex life. Most were single, without partners at the time of the survey. Among women, sex life is more restricted than among men. The patients’ sexual life shows a development similar to previous studies, in which the postponement of the beginning of sexual life or its absence (both in XX DSD and XY DSD), may be related to other aspects of the individual’s life, such as body image, self-esteem, or feelings of shame or fear.

Sexual attraction to both men and women (homosexuality, heterosexuality, bisexuality), as well as the presence of masturbatory acts, indicate the presence of sexual desire directed toward the other. This aspect points to the need for continuous information and guidance on the sexual aspect, safeguarding the individual differences of every person.

Finally, the sample limitation does not prevent us from observing, in an exploratory way, that the DSD condition involves not only physical and genital development, but also subjective conditions, such as sexual functionality, both in the individual sphere and in the relationship with others.

## Data Availability Statement

The raw data supporting the conclusions of this article will be made available by the authors, without undue reservation.

## Ethics Statement

The studies involving human participants were reviewed and approved by UFRGS—Hospital de Clínicas de Porto Alegre da Universidade Federal—Plataforma Brasil. Written informed consent to participate in this study was provided by the participants’ legal guardian/next of kin.

## Author Contributions

All authors listed have made a substantial, direct, and intellectual contribution to the work and approved it for publication.

## Conflict of Interest

The authors declare that the research was conducted in the absence of any commercial or financial relationships that could be construed as a potential conflict of interest.

## Publisher’s Note

All claims expressed in this article are solely those of the authors and do not necessarily represent those of their affiliated organizations, or those of the publisher, the editors and the reviewers. Any product that may be evaluated in this article, or claim that may be made by its manufacturer, is not guaranteed or endorsed by the publisher.
